# Auditory screening in the elderly: comparison between self-report and audiometry

**DOI:** 10.1016/S1808-8694(15)31310-0

**Published:** 2015-10-20

**Authors:** Cláudia Maria Valete-Rosalino, Suely Rozenfeld

**Affiliations:** ^1^ Physician, Post-graduation in Otorhinolaryngology, Specialist in Otorhinolaryngology, Master in Otorhinolaryngology; Ph.D. studies in Public Health (Technologist/Professor) - Service of Otorhinolaryngology, Instituto de Pesquisas Clínicas Evandro Chagas - FIOCRUZ; Department of Otorhinolaryngology and Ophthalmology – Federal University of Rio de Janeiro; Ph.D. studies under course, Escola Nacional de Saúde Pública – FIOCRUZ; ^2^ Physician, Master and Ph.D. in Public Health (Researcher) - Department of Epidemiology and Health Quantitative Methods - Escola Nacional de Saúde Pública – FIOCRUZ

**Keywords:** hearing loss, aged, questionnaires, audiometry, validity

## Abstract

Despite its high prevalence in the aged, hearing loss has been poorly investigated. Audiometry is the gold standard for evaluation of hearing loss, but large-scale use of the procedure involves operational difficulties. Thus, self-report may be an alternative.

**Aim:**

To determine if a single global question is valid for use in epidemiologic research.

**Study design:**

Systematic review.

**Material and Method:**

A search of the medical literature from 1990 to 2004 was performed using MEDLINE and LILACS. The references of the articles identified in the electronic search were also reviewed. **Study Selection and Data Extraction**: The articles that compared the results obtained with self-report to a single global question with those obtained by pure tone audiometry were selected. Data about the prevalence of hearing loss, and sensitivity, specificity and predictive values were extracted. **Data Synthesis**: Ten longitudinal studies were included. A single global question seems to be an acceptable indicator of hearing loss, sensitive and reasonably specific, mainly if the hearing loss is identified as the tone average that includes frequencies up to 2 or 4 kHz, at 40 dBHL level, in the best ear.

**Conclusion:**

A single global question shows good performance in identifying older persons with hearing loss and can be recommended for an epidemiologic study if audiometric measurements cannot be performed.

## INTRODUCTION

Considering the age of 60 years as the dividing line between elderly and non-elderly, we observed based on IBGE data (Brazilian Institute of Geography and Statistics) that there has been percentage increase in number of elderly people (60 years and over) in Brazil, from 5.07% in 1970 to 8.56% in 2000^1^. The number of elderly patients went up from 3 million in 1960 to 7 million in 1975, to 14 million in 2002 (a 500% increase within 40 years). It is estimated that it will reach 32 million by 2020^2^. If there are further advances in mortality drop at advanced ages, this process will be even further enhanced^1^.

Among pathologies whose frequency increases with aging, we can include those related with inner ear. Hearing loss is the third most prevalent chronic condition among American elderly patients, third to hypertension and arthritis^3^. In Brazil, studies have shown a prevalence of hearing loss in the elderly that ranges from 20 to 85%^4^, ^5^, ^6^, ^7^. Hearing loss has been associated with negative psychosocial impact^8^, with inability to perform heavy home chores^9^ and increase in occupational accidents^10^. Auditory sensorial loss not corrected by hearing aids is associated with loss of self-sufficiency in daily living activities and social relationship impairment in the elderly, plus increased mortality in males^11^. The wish to wear a hearing aid is not associated with severity of loss, but rather with functional status of the subject, being higher among more independent subjects^8^.

Despite the high prevalence among elderly patients, hearing loss is one of the problems not investigated during routine medical examination in this age range^12^. Screening can be useful in identifying primary health care, given that the onset is insidious and that patients are frequently unaware of it. Audiometry is the gold standard test, but its conduction is sometimes hindered in some regions owing to problems of access, reference and reimbursement. Thus, many clinicians rely on self-administered questionnaires^13^. As to research studies, large-scale trials of auditory status may provide clues on temporal trends of prevalence of hearing loss and contribute to the identification in geographical areas and subgroups of risk populations (gender, race and ethnicity). These investigations may be a quick and cheap way of providing estimates to large populations, in which expenses (audiometric equipment and trained personnel) and time restrictions are a concern^14^. Generic isolated issues on hearing have also been used in epidemiological studies. Self-report can also be an indicator of hearing loss and it is quick and cheap to administer^15^.

Considering that audiometry is the gold standard for hearing loss detection and requires trained team, soundproof booth and equipment, hindering its execution in large scale, our final goal was to determine whether one single global question would be valid to be used in epidemiological research studies. This paper aimed at reviewing studies whose purposes were to compare results obtained with use of isolated questions and results obtained through pure tone audiometry in assessment of hearing loss in the elderly.

## MATERIAL AND METHOD

### Identification and selection of studies

We conducted a literature review on databases MEDLINE and LILACS, in the period between 1990 and 2004 to retrieve articles comparing results obtained in the assessment of hearing loss in the elderly through self-report using one single global question and pure tone audiometry. In the electronic search we used the terms *hearing loss, hearing impairment, deafness, presbycusis, questionnaires, self-report, question, validity, audiometry*, isolated or in combination and accepted texts in all languages. We also analyzed articles cited in the references of other articles identified in the electronic search. We selected 10 articles from MEDLINE and none from LILACS. We excluded one article because it did not provide isolated data on validity of the use of one single global question on hearing loss assessment compared to pure tone audiometry^16^, one article that did not specify the results in the elderly^17^, and another one that did not compare the results of one single question with audiometry^18^.

### Data extraction

We extracted data on prevalence of hearing loss observed by audiometry, estimated loss by the question and difference between prevalence of observed and estimated hearing loss. Sensitivity, specificity and predictive values of hearing loss assessment through one single global question in comparison to audiometry were also included. One study presented estimate of odds ratio (OR) of the question concerning hearing loss measured by audiometry and another one assessed the association between the question and the mean thresholds in different frequencies.

### Characteristics of analyzed studies

In Table 1 we can see the description of general characteristics of analyzed studies. We located 10 transversal studies and seven had population basis^13^, ^14^, ^15^, ^19^, ^20^, ^21^, ^22^, one was conducted on nursery home patients^23^, one on workers in a technology company^24^ and one was hospital-based^8^. Studies involved samples that ranged from 198 to 12,495 subjects, except for one study that included only 63 subjects^8^. All studies included subjects aged 60 years or more, and one of them included only women^20^.

**Table 1:** General characteristics of validation studies of the hearing loss questionnaire for the elderly.

Author, year, country	Sample	Criteria of hearing loss for pure tone audiometry	Question
Gates et al., 1990^19^USA	N = 1662 Age: 63-95 years; Mean: 73 years Gender: 41% (M) and 59% (F)	MT 0,5, 1, 2 kHz > 25 dB NA, best ear MT 0,5, 1, 2 and 3 kHz > 25 dB NA, best ear	Dou you have a hearing problem now?
Clark et al., 1991^20^USA	N = 267 Age: 60-85 years Gender: 100% (F)	MT 1 and 2 kHz or 1, 2, 3 and 4 kHz ≥25 dB HL or ≥40 dB HL, best ear and worst ear	Would you say that you have any difficulty hearing?
Voeks et al., 1993^23^USA	N = 198 Age: NI; Mean: 72.4 ± 11.4 years Gender: 81.8% (M) and 18.2% (F)	MT 0,5, 1 and 2 kHz > 25 dB HL, best ear thresholds 1 to 2 kHz > 40 dBHL in both ears or thresholds 1 and 2 kHz > 40 dBHL in one ear	Do you have trouble hearing?
Reuben et al., 1998^21^USA	N = 917 Age: 55-74 years Gender: NI	MT 1, 2 e 4 kHz e”25 dbNA, best ear	Have you ever had deafness or trouble hearing with one or both ears?
Nondahl et al., 1998^14^ USA	N = 3556 Age: 48-92 years; Mean: 65.8 years Gender: 42.3% (M) and 57.7% (F)	MT 0,5, 1, 2 and 4 kHz > 25 dB HL, worst ear	1. Do you feel you have a hearing loss? 2. In general, would you say your hearing is (1) excellent, (2) very good, (3) good, (4) fair, (5) poor
Sindhusake et al., 2001^15^Australia	N = 2015 Age: 55-100 years Gender: 42.6% (M) and 57.4% (F)	MT 0,5, 1, 2 and 4 kHz > 25 dB HL, > 40 dB HL and > 60 dB HL, best ear	Do you feel you have a lhearing oss?
Gates et al., 2003^13^USA	N = 546 Age: 72-94 years; Mean: 78.3 ± 4.1 years Gender: 35.5% (M) and 64.5% (F)	thresholds 1 or 2 kHz ≥40 dBHL in both ears or thresholds 1 and 2 kHz ≥40 dBHL in one ear	Do you have a hearing problem now?
Uchida et al., 2003^22^Japan	N = 2150 Age: 40-79 years N = 539: 50-59 years; N = 544: 60-69 years; N = 529: 70-79 years Gender: 51.3% (M) and 48.7% (F)	thresholds 0,5, 1, 2, 4 and 8 kHz best ear and worst ear	Do you feel you have hearing loss?
Hashimoto et al., 2004^24^ Japan	N =12.495 Age: over 30 years; Mean: 47.8 ± 7.0 N = 5.095: 50-59 years; N = 343: over 60 years Gender: 92.7% (M) and 6.3% (F)	MT 1 and 4 kHz ≥25 dBHL, Best ear	Do you have difficulty in hearing? 0 = no hearing problem; 1 = same as before; 2 = progressive; 3 = getting worse
Wu et al., 2004^8^ Singapore	N = 63 Age: 62-90 years; Median: 79 years Gender: 39.7% (M) and 60.3% (F)	thresholds 1 or 3 kHz, > 30 dB HL or > 50 dB HL, one ear	Do you think you have a hearing problem?

NI = not informed; (M) = male; (F) = female; MT = pure tone mean; kHz = frequency in KHertz; dB HL = dB hearing level.

Pure tone audiometry criteria varied according to frequency, intensity and assessed ears. Seven studies assessed pure tone mean (MT), with variation of frequencies used in the calculation between 0.5, 1, 2, 3 and 4 kHz^14^, ^15^, ^19^, ^20^, ^21^, ^23^, ^24^. Three studies used individual thresholds of frequencies, and two of them were 1 and 2 kHz^13^, ^21^ and the other was 1 and 3 kHz^8^. One study used isolated thresholds of frequencies 0.5, 1, 2, 4 and 8 kHz and compared means of thresholds of each frequency with self-report of hearing loss^22^. Intensity of stimulus used as cut-off point was 25dB hearing level (dBHL) in seven studies^14^, ^15^, ^19^, ^20^, ^21^, ^23^, ^24^. Four studies used 60dB HL as cut-off point^15^ and the other one used intensities of 30 and 50 dB HL^8^. Seven studies used the best ear in the assessment of hearing loss^15^, ^19^, ^20^, ^21^, ^22^, ^23^, ^24^ and three used the worst ear^14^, ^20^, ^22^. Three studies considered both ears or the worst ear^8^, ^13^, ^21^.

The questions used were similar one to the other, and five studies used questions with yes-no questions^8^, ^13^, ^19^, ^20^, ^21^, two considered the answered “I don't know” and excluded it from the study^14^, ^15^, one considered the answer “I don't know” as positive to the hearing loss and analyzed it together with “yes”^23^, one considered the response “occasionally” together with “yes” in the analysis of estimated prevalence of hearing loss, but considered it as isolated concerning the other assessments^22^. Other two studies used multiple response questions, and Hashimoto et al. (2004)^24^ considered as negative to the hearing loss only the option “no hearing problem”, whereas Nondahl et al. (1998)^14^ considered as positive to the hearing loss the options “fair” and “poor”.

### Synthesis of Data in Analyzed Studies

Table 2 presents observed and estimated prevalence, sensitivity, specificity and predictive values found in different studies.

**Table 2:** Prevalence of hearing loss and validity of one single question questionnaire.

Author, Year	Observed prevalence of hearing loss by pure tone audiometry (%) Estimated prevalence of hearing loss by question (%) Difference between observed and estimated hearing loss (%)	Sensitivity (%) and Specificity (%)	Positive Predictive Value (%) and Negative Predictive Value (%)
Gates et al., 1990^19^	PO (0,5-2 kHz) = 29 e PO (0,5-3 kHz) = 42 PE = 41 PO-PE (0,5-2 kHz) = -12 and PO-PE (0,5-3 kHz) = 1	S (0,5-2 kHz) = 89,93 E (0,5-2 kHz) = 86,93	VPP (0,5-2 kHz) = 79,80 VPN (0,5-2 kHz) = 93,77
Clark et al., 1991^20^	PO - Best ear ≥25 dBNA: (1-2kHz) = 34 and (1-4kHz) = 45 ≥40 dBNA: (1-2kHz) = 11 and (1-4kHz) = 18 PO- Worst ear ≥25 dBNA: (1-2kHz) = 42 and (1-4kHz) = 60 ≥40 dBNA: (1-2kHz) =18 and (1-4 kHz) = 27 PE = 35	Best ear ≥25 (1-2kHz): S = 66; E = 80 ≥25 (1-4kHz): S = 56; E = 82 ≥40 (1-2 kHz): S = 90; E = 71 ≥40 (1-4 kHz): S = 83; E = 75	Best ear ≥25 (1-2kHz): VPP = 63; VPN = 82 ≥25 (1-4kHz): VPP = 71; VPN = 71 ≥40 (1-2 kHz): VPP = 28; VPN = 98 ≥40 (1-4 kHz): VPP = 42; VPN = 96
	PO-PE - Best ear ≥25 dBNA: (1-2kHz) = -1 and (1-4kHz) = 10 ≥40 dBNA: (1-2kHz) = -24 and (1-4kHz) = -17 PO-PE - Worst ear ≥25 dBNA: (1-2kHz) = 7 and (1-4kHz) = 25 ≥40 dBNA: (1-2kHz) = -17 and (1-4 kHz) = -8	Worst ear ≥25 (1-2kHz): S = 58; E = 82 ≥25 (1-4kHz): S = 51; E = 88 ≥40 (1-2 kHz): S = 81; E = 74 ≥40 (1-4 kHz): S = 70; E = 77	Worst ear ≥25 (1-2kHz): VPP = 70; VPN = 73 ≥25 (1-4kHz): VPP = 86; VPN = 43 ≥40 (1-2 kHz): VPP = 40; VPN = 95 ≥40 (1-4 kHz): VPP = 54; VPN = 87
Voeks et al., 1993^23^	PO = 54 PE = 60 PO-PE = -6	S = 69,2 E = 50,6	VPP = 62,2 VPN = 58,2
Reuben et al., 1998^21^	PO > 40 dBNA (1-2 kHz) = 14,2 PO ≥25 dBNA (1-4 kHz) = 35,1 PE = 24 PO-PE > 40 dBNA (1-2 kHz) = -9,8 PO-PE ≥25 dBNA (1-4 kHz) = 11,1	-	-
Nondahl et al., 1998^14^	PO = 45,9, PE(Q1) = 47,8 and PE(Q2) = 24,2 PO-PE(Q1) = -1,9 and PO-PE(Q2) = 21,7 PO-PE(Q1) Best ear = -14,8	S(Q1) = 71 and E(Q1) = 71 S(Q2) = 43 and E(Q2) = 93	VPP(Q1) = 68 and VPN(Q1) = 74 VPP(Q2) = 83 and VPN(Q2) = 66
Sindhusake et al., 2001^15^	PO > 25 dBNA = 40, PO > 40 dBNA = 14 and PO > 60 dBNA = 2 PE = 51 PO-PE > 25 dBNA = -11, PO-PE > 40 dBNA = -37 and PO-PE > 60 dBNA = -49 PO-PE > 25 dBNA Worst ear = 2,3	>25 dBNA: S = 78 and E = 67 >40 dBNA: S = 93 and E = 56 >60 dBNA: S = 100 and E = 50 >25 dBNA Worst ear: S = 71 and E = 72	>25 dBNA: VPP = 61 e VPN = 82 >40 dBNA: VPP = 25 e VPN = 98 >60 dBNA: VPP = 5 e VPN = 100 > 25 dBNA Worst ear: VPP = 71 and VPN = 69
Gates et al., 2003^13^	PO = 27, PE = 40 and PO-PE = -13	S = 71 and E = 72	VPP = 48 and VPN = 87
Uchida et al., 2003^22^	PE: 50-59 years = 43,4, 60-69 years = 49,4 and 70-79 years = 56,1	-	-
Hashimoto et al., 2004^24^	PO-Best ear: 50-59 years = 7,1 and ≥60 years =14,9 PO-Worst ear: 50-59 years = 19,8 and ≥60 years = 30,9 PE: 50-59 years = 6,2 and ≥60 years = 6,1 PO-PE Best ear: 50-59 years = 0,9 and ≥60 years = 8,8 PO-PE Worst ear: 50-59 years = 13,6 and ≥60 years = 24,8	50-59 years: S = 23 and E = 95 ≥60 years: S = 14 and E = 95	50-59 years: VPP = 26 and VPN = 94 ≥60 years: VPP = 33 and VPN = 86
Wu et al., 2004^8^	PO = 83, PE = 49,2 and PO-PE = 33,8	S = 58 and E = 91	VPP = 97 and VPN = 31

PO = Observed prevalence of hearing loss by pure tone audiometry; PE: Estimated prevalence of hearing loss by question; PO-PE: Difference between observed and estimated hearing loss; S = sensitivity; E = specificity; VPP = positive predictive value; VPN = negative predictive value; Q1 = question 1; Q2 = question 2.

Observed prevalence by pure tone audiometry ranged from 2% (> 60 dBHL^15^) to 83%^8^. Excluding the study by Hashimoto et al. (2004)^24^, who studied subjects over the age of 30 years, the observed prevalence was higher when using cut-off intensity of 25 dBHL (29 to 60%) than when using cut-off points of 40 dBHL (11 to 27%) and 60 dBHL (2%). Excluding the analysis of cut-off point of 60dBHL, studies assessed up to the frequency of 2kHz presented lower prevalence values (11 to 54%) than the ones that assessed up to 4 kHz (18 to 60%). Considering the assessed ear, the best ear presented lower values of observed prevalence (11 to 54%) than the worst ear (18 to 60%). Estimated prevalence ranged from 6.1% (subjects aged over 60 years by Hashimoto et al., 2004) to 60%^23^. In absolute numbers, the difference in observed and estimated prevalence ranged from 0.9 (50-59 years by Hashimoto et al., 2004) to 49 (> 60 dBHL^15^). In other words, the observed prevalence increased with reduction of intensity cut-off point, with increase of studied frequency and when they considered the worst ear.

Sensitivity ranged from 14% (over the age of 60 years^24^) to 100% (> 60 dBHL^15^) and specificity ranged from 50% (> 60 dBHL^15^) to 95% (over the age of 60 years^24^). Positive predictive value (PPV) ranged from 5% (> 60 dBHL^15^) to 97%^8^ and negative predictive value (NPV) ranged from 31%^8^ to 100% (> 60 dBHL^15^). We observed that sensitivity of both questions with multiple-choice responses was lower, and for this reason we did not include their results in the analysis that follows. As to studied frequencies, Clark et al. (1991)^20^ were the only ones that presented results with two distinct criteria owing to used frequencies of hearing loss through pure tone audiometry. The results suggested that the use of pure tone mean of frequencies 1, 2, 3 and 4 kHz presented lower values of sensitivity and NPV and higher values of specificity and PPV, comparing the pure tone mean of frequencies 1 and 2 kHz. Sensitivity was higher when using 40 dBHL as cut-off point (70 to 93%) and it was lower when the cut-off point was 25 dBHL (51 to 89.9%). Conversely, specificity was higher using the cut-off point 25 dBHL (50.6 to 88%) than using 40 dBHL (56 to 77%). As to used ear, the best ear had higher values of sensitivity (56 to 100%) than the worse ear (51 to 81%), and the worst ear had higher values of specificity (71 to 88%) than the best ear (50 to 86.9%). PPV was higher when using 25 dBHL (62.2 to 86%) as cut-off point and it was lower when the cut-off point was 40 dBHL below (25 to 54%). NPV was higher when using 40 dBHL (87 to 98%) as cut-off point and it was lower when the cut-off point was 25 dBHL lower (43 to 93.8%). As to used ear, the best ear had the highest values of negative predictive value (58.2 to 98%) than the worst ear (43 to 95%), and the worst ear took to higher positive predictive value (40 to 86%) than the best ear (25 to 79.8%). The only study that used cut-off point at 60 dBHL presented the highest values of sensitivity and NPV (100%) and the lowest values of specificity (50%) and PPV (5%).

Gates et al. (1990)^19^ referred that the proportion of male that detected hearing problem (50%) was higher than that of female subjects (35%) (Ç^2^ = 38.58, p < 0.0001). To both men and women, prevalence of self-reported hearing impairment increased with each 5-year increase in age group (women: Ç^2^ = 57.2, p < 0.001; men: Ç^2^ = 18.1, p = 0.02). Pure tone mean in the best ear in 683 patients that reported an auditory problem was significantly worse than in 979 cases that did not report hearing problems. Pure tone mean in the best ear was significantly higher among men (MT 0.5-2 kHz = 22.0 ± 0.52; MT 0.5-3 kHz = 27.9 ± 0.53) than among women (MT 0.5-2 kHz = 20.4 ± 0.42; MT 0.5-3 kHz = 22.7 ± 0.43). As a result of increased age, there is generalized worsening of thresholds in all frequencies, especially in higher ones. Authors detected a significant difference (Ç^2^ = 6.23, p = 0.013) between proportion of men (32.5%) and women (26.7%) that were classified as having hearing loss with MT 0.5-2 kHz.

Reuben et al. (1998)^21^ observed in people with positive self-report to hearing loss a chance almost 10 times higher (OR = 9.8, CI: 7.8-12.4) of having a hearing loss compared to people whose self-report was negative, according to the criteria of thresholds 1 or 2 kHz > 40 dBHL in both ears or thresholds 1 and 2 kHz > 40 dBHL in one ear, and almost five times higher (OR = 4.8, CI: 4.0-5.9) according to criteria of pure tone mean of 1, 2 and 4 kHz e•25 dBHL in the best ear. Observed prevalence of hearing loss was significantly higher in men, in the older groups and when cut-off criterion was at 25 dBHL.

Nondahl et al. (1998)^14^ checked the presence of 71% accuracy in question 1 (binary response) and accuracy of 70% in question 2 (multiple category of responses). They observed that questions had sensitivity and PPV that were higher in men and specificity, NPV and general accuracy that were higher in women. As to age, they observed that questions had greater sensitivity in the younger group (48-64 years), as well as in most cases they also presented higher accuracy and better estimate of prevalence in the group.

Sindhusake et al. (2001)^15^ reported results of sensitivity and specificity that were separated by gender and age and observed that they were minimally affected. The question presented higher sensitivity and lower specificity in male subjects, and higher sensitivity and specificity in the younger group (below 70 years).

Uchida et al. (2003)^22^ observed that prevalence of self-reported hearing loss was significantly higher in older subjects and in both genders and that it was higher in women aged 40 to 59 years. They also observed statistically significant correlation between self-report of hearing loss and pure tone thresholds on the best and worst ears in both age groups. In other words, thresholds in the same age range and in the same frequency were significantly higher in relation to the three groups of respondents (“yes”, “occasionally” and “no“), and the thresholds in the group that responded “yes” were higher than those that responded “occasionally”, which in turn were higher than those that responded “no”. They also observed significantly higher thresholds in men and each age range with increase of 10 years in each group of respondents.

Hashimoto et al. (2004)^24^ reported agreement between self-report of hearing loss and hearing loss criteria by pure tone audiometry in 90% in the age range 50-59 years and 83% after the age of 60 years.

## DISCUSSION

The present study aimed at comparing the prevalence estimates of hearing loss obtained using two methods: self-report and audiometry. According to the analysis of the studied literature, the single global question seems to be an acceptable indicator of hearing loss which is sensitive and reasonably specific, especially when the loss is identified as being pure tone mean up to frequencies 2 or 4 kHz, at 40 dBHL on the best ear.

The comparison of hearing loss prevalence among the studies is hindered by the differences in investigated populations and in audiometric criteria used for its definition^14^, ^20^. Prevalence of hearing loss is smaller in women and younger people^20^. Moreover, elderly people selected from a nursing home^23^ or hospital setting^8^ represented a fragile selected group, presenting more comorbidities than healthy elderly people from the general population, leading to increase in prevalence of hearing loss in comparison to population-based studies^8^, ^20^. The discrepant results found by Hashimoto et al. (2004)^24^ and the other studies may be related to the population studied by the former, which comprised industry workers. Therefore, part of the studied people could have preferred to say they did not have a hearing loss in fear of losing their jobs, malingering the audiometric performance. In addition, there is also the possibility that the results had been affected by the healthy-worker effect, even though the prevalence had been comparable to that of other Japanese studies^24^.

Definitions of hearing loss assessed by pure tone mean in the literature range according to the ear used to classify the subject (i.e., best, worst, both, right or left ear) and to frequencies included in the pure tone mean to determine the best and the worst ear^22^. The use of worst ear to define hearing loss results increased prevalence^14^. Inclusion of frequency of 4 kHz has also increased prevalence of hearing loss^14^, ^23^ given that frequencies 0.5 to 2 kHz are considered important in the identification of the disability related with hearing, frequencies 3 and 4 kHz are among the first ones to show hearing decrease associated with age and they are important for speech understanding, especially in noisy environment^20^. Even though many different definitions of hearing loss had been proposed, there is no universal acceptance, and stricter definition (low intensity as cut-off point, such as 25 dBHL) leads to higher prevalence of hearing loss^19^.

As to self-report of hearing loss comparing to pure tone audiometry classification criteria, the use of the best ear is justified by the fact that the worst ear tends to be compensated by the function of the side that has the best subjective perception^24^. Self-perception of hearing loss seems to be in agreement with pure tone mean of medium frequencies (1, 2, 3 and 4 kHz)^16^ and it is more frequent in subjects with moderately-severe hearing loss than among those with mild loss^8^, ^23^.

As to questions used, multiple-choice questions^14^, ^24^ presented lower sensitivity when compared to two-option questions. The type of question used by Hashimoto et al. (2004)^24^ may have caused confusion given that the answer “same as before” may have been used to mean “no hearing problem since then”, even though when they classified “same as before” as having no hearing loss, there were no statistically significant differences in sensitivity and specificity of the question. Moreover, even though two-option questions had been similar one to the other, such as those used by Gates et al. (1990^19^ and 2003^13^), they may have given the impression to respondents that they were more serious difficulties, which could have reduced the estimated prevalence of hearing loss^14^.

Voeks et al. (1993)^23^ considered mistaken the response “I don't know” as a positive indication of hearing loss because they consider that this strategy conveys more sensitivity to the question, whereas other studies considered them as missing values^14^, ^15^. However, in the group studied by Voeks et al. (1993)^23^ there were some subjects with cognitive deficit, which could have hindered the identification through a questionnaire and reduced sensitivity, given that among the three above-referred studies, the lowest sensitivity was reached by Voeks et al. (1993)^23^. In addition, the study by Voeks et al. (1993)^23^ detected more wrong responses in the group without hearing loss in the audiometry, increasing the number of false positives and justifying the low specificity found by them. They also referred that the response “yes” conveys certainty about the hearing loss, whereas “I don't know” response conveys a little more than 50% certainty^23^.

According to Hashimoto, Nomura and Yano (2004)^24^, simple questions such as “Do you feel you have a hearing loss?” are based on subjective assessment of individual health status, and they may be distorted by other psychosomatic symptoms and mental health status. They observed higher proportion of false positives in those with more than two complaints of somatic symptoms, and wondered whether these patients would not be the most sensitive ones to all somatic sensations - they could have exaggerated about their hearing acuity. Thus, it might be reasonable to say that subjective hearing difficulties without objectively diagnosed impairment may reflect psychosocial problems experienced in daily communication situations at the workplace.

Screening approaches try to increase the likelihood a person with a specific dysfunction has to be identified (sensitivity), excluding those without the dysfunction (specificity). In practice, not all cases are identified (false negative), and some people without dysfunction will be misdiagnosed (false positive)^13^. Therefore, the more sensitive the test is for hearing loss, the higher the likelihood of false positives, and among those, many might have had some degree of dysfunction, even if they had not reached the specific criteria set by audiometry, benefiting from the referral to complete audiometric assessment^13^. Hashimoto et al. (2004)^24^ referred that discrepancy of results in relation to other studies should be related with high number of false negative results. Given that subjects in the study were relatively younger than the other studied subjects and that normal speech is produced at frequencies of about 1 kHz, even if the studied subjects had early signs of sensorineural hearing loss, they would not have been recognized in daily verbal communication. They explained the number of false negative results in the study by the fact that the proportion of subjects that complained of auditory difficulties was smaller in the group of people that had hearing loss only in the frequency of 4 kHz, compared to the group whose affection comprised 1 and 4 kHz. They reported that since audiometry was conducted yearly, they might have experienced some learning bias, and in addition, people might have stated that they had auditory difficulties because they remembered the results of hearing loss detected by previous year audiometry. It may artificially reduce false negative results and actual sensitivity could be even lower^24^.

As to results of the studies based on gender and age, Gates et al. (1990)^19^ warned to the possible role of noise exposure as basis for different etiology between men and women, whereas Uchida et al. (2003)^22^ observed that men tended to underestimate their hearing difficulty more than women. Sindhusake et al. (2001)^15^ did not find statistically significant differences concerning gender and age, and Nondahl et al (1998)^14^ did not describe whether the differences found by them had been statistically significant. The greater sensitivity to the question found in the younger group in these two studies^14^, ^15^, might have been explained by low self-perception or denial of problems faced by the elderly^8^, considering that there is a popular belief that hearing loss is a normal part of aging and not a health problem that deserves special attention^14^, making them underestimate their auditory difficulty^22^.

As to both studies that did not present results of sensitivity, specificity and predictive values, Reuben et al. (1998)^21^ conducted a study in the 70's and the traditional trend poses a risk to generalization of data on prevalence and validity of the study, because they used a relatively young sample, maximum age of 74 years, which should have reduced the number of subjects with positive screening. Uchida et al. (2003)^22^ obtained reasonable performance in the question used by them to stratify subjects by hearing loss level.

Gates et al. (1990)^19^ stated that hearing is poorly described through one single parameter, be it self-report or pure tone thresholds, and it is highly prevalent among elderly people and increases in older groups. Few studies with representative population samples used audiometry as a hearing assessment method. A population-based study allows generalization of results, provided that geographical areas, ethnics and noise exposure levels are the same. Studies based on convenience or clinical samples, with or without estimates derived from other geographical regions, may lead to false results concerning hearing loss. If the purpose of hearing loss measurement is to associate it with other factors, then knowledge of characteristic of mistakes comparing questionnaire and audiometry through validation study is quite convenient.

We should also add that we did not find any Brazilian studies that compared one single question questionnaire to pure tone audiometry, and we do not have estimates that can be generalized to the Brazilian population.

## CONCLUSION AND SUGGESTIONS

One single global question has good performance to identify elderly people with hearing loss and it may be recommended for epidemiological studies that cannot apply audiometric measurements. Considering that we do not have estimates that can be generalized to the Brazilian population, it would be recommendable to conduct a validation study of one single global question compared to audiometry, allowing the use of this useful tool in Brazilian epidemiological studies, with the advantage of being able to study a large number of subjects.
